# Supplementary material for: Predicting evolutionarily stable strategies from functional responses of Sonoran Desert annuals to precipitation
Source: Proc Biol Sci. 2019 Jan 16;286(1894):20182613. doi: 10.1098/rspb.2018.2613 (PMC6367162; doi:10.1098/rspb.2018.2613)
Supplement: Appendix A123 [file rspb20182613supp1.pdf]

Appendix

A1 - ESS Calculations

If we let $Z(\tilde{g}) = \left(\frac{\tilde{g}K_1 s_{\text{new}}}{1+ag\hat{n}(g)} + (1-\tilde{g})s_{\text{old}}\right)$, then

$$\frac{\partial r}{\partial \tilde{g}}(g,g) = \mathbb{E}\left[\frac{\left(\dfrac{K_1 s_{\text{new}}}{1+ag\hat{n}(g)} - s_{\text{old}}\right)}{Z(g)}\right] \tag{1}$$

where the expected value is evaluated with respect to $\hat{n}(g)$ and K_1. Letting $h(g) = \frac{\partial r}{\partial \tilde{g}}(g,g)$, it is sufficient to solve for the unique zero of h, if it exists, over the domain $(0,1)$. To accomplish this, we take 2,000 draws from $\hat{n}(g)$, evaluate each of these draws via expression (2) of the main text, and average the corresponding outputs. Using R's uniroot solver, we then solve for when $h(g) = 0$.

A2 - Nondimensionalization

Let $x_t = an_t$, where $a > 0$. Then substituting into (1) of the main text, we obtain

$$x_{t+1} = \frac{gK_{t+1}s_{\text{new}}x_t}{1+gx_t} + (1-g)s_{\text{old}}x_t. \tag{2}$$

Since a does not appear in (3), solving for the ESS value depends only on whether $a = 0$ or $a > 0$, rather than the specific value of a non-zero competition coefficient. Since all of our a values are positive, we can rule this out as a predictor.

A3 - System Design

Long-term demographic monitoring of the Sonoran Desert winter annual community has been conducted since 1982 at the University of Arizonas Desert Laboratory at Tumamoc Hill in Tucson, AZ ($32°13'$ N, $111°0'$ W), 723 m above sea level. Every year since then, per-

manently marked plots along a 250m transect have been visited to record germination of every plant in each plot and then surveyed at roughly monthly intervals to monitor survival. Beginning in the 1989/1990 season, the density of viable, non-germinating seeds were estimated using soil cores collected outside of plots but within the demographic study area. Combined with the demographic surveys, these seed bank estimates were used to calculate annual germination fractions. Details on the demographic and seed bank studies are available, but kept brief here [1–4]. To be consistent with previous work [2, 3], we analyzed the data for 10 common and abundant species in the winter annual community: *Eriophyllum lanosum A. Gray* (ERLA, Asteraceae), *Erodium cicutarium (L.) L'Hér. ex Aiton* (ERCI, Geraniaceae; naturalized species), *Erodium texanum A. Gray* (ERTE, Geraniaceae), *Evax multicaulis DC* (EVMU, Asteraceae), *Monoptilon bellioides (A. Gray) H.M. Hall* (MOBE, Asteraceae), *Pectocarya recurvata I. M. Johnst.* (PERE, Boraginaceae), *Plantago patagonica Jacq.* (PLPA, Plantaginaceae), *Plantago insularis Forssk.* (PLIN, Plantaginaceae), *Schismus barbatus (Loefl. ex L.) Thell.* (SCBA, Poaceae; naturalized species), and *Stylocline micropoides A. Gray* (STMI, Asteraceae). Here we use the nomenclature consistent with previous work on this system; synonymy and currently accepted nomenclature are given in Gremer et al. [4].

References

[1] C. E. Pake and D.L. Venable. Seed banks in desert annuals: implications for persistence and coexistence in variable environments. *Ecology*, 77(5):1427–1435, 1996.

[2] D.L. Venable. Bet hedging in a guild of desert annuals. *Ecology*, 88(5):1086–1090, 2007.

[3] J.R. Gremer and D.L. Venable. Bet hedging in desert winter annual plants: optimal germination strategies in a variable environment. *Ecology Letters*, 17(3):380–387, 2014.

[4] J.R. Gremer, S. Kimball, and D.L. Venable. Within-and among-year germination in

sonoran desert winter annuals: bet hedging and predictive germination in a variable environment. *Ecology Letters*, 19(10):1209–1218, 2016.
